# Modulation Doping of Silicon using Aluminium-induced Acceptor States in Silicon Dioxide

**DOI:** 10.1038/srep46703

**Published:** 2017-04-20

**Authors:** Dirk König, Daniel Hiller, Sebastian Gutsch, Margit Zacharias, Sean Smith

**Affiliations:** 1 Integrated Materials Design Centre (IMDC), UNSW, Sydney, Australia; 2 School of Photovoltaic and Renewable Energy Engineering (SPREE), UNSW, Sydney, Australia; 3 Laboratory for Nanotechnology, Dept. of Microsystems Engineering (IMTEK), University of Freiburg, Germany

## Abstract

All electronic, optoelectronic or photovoltaic applications of silicon depend on controlling majority charge carriers via doping with impurity atoms. Nanoscale silicon is omnipresent in fundamental research (quantum dots, nanowires) but also approached in future technology nodes of the microelectronics industry. In general, silicon nanovolumes, irrespective of their intended purpose, suffer from effects that impede conventional doping due to fundamental physical principles such as out-diffusion, statistics of small numbers, quantum- or dielectric confinement. In analogy to the concept of modulation doping, originally invented for III-V semiconductors, we demonstrate a heterostructure modulation doping method for silicon. Our approach utilizes a specific acceptor state of aluminium atoms in silicon dioxide to generate holes as majority carriers in adjacent silicon. By relocating the dopants from silicon to silicon dioxide, Si nanoscale doping problems are circumvented. In addition, the concept of aluminium-induced acceptor states for passivating hole selective tunnelling contacts as required for high-efficiency photovoltaics is presented and corroborated by first carrier lifetime and tunnelling current measurements.

Conventional silicon doping is increasingly impeded due to the spatial dimensions approached by nanotechnology. Several fundamental physical principles counteract the substitutional incorporation of dopant impurities (e.g. B, P, or As) on Si lattice sites and their ionization to become electronically active donors of majority charge carriers. The formation energy for the substitutional dopant integration^1^,^2^ as well as the ionization energy^3^,^4^,^5^,^6^ increase strongly with decreasing Si dimensions. Moreover, conventional doping of Si nanoscale devices faces severe technological challenges: diffusion-related, steep radial gradients in the doping profile of Si nanowires^7^,^8^,^9^; the inadvertent but inevitable diffusion of source/drain dopants into field-effect transistor (FET)-channels^10^; surface segregation and inactivation of dopants^11^; and statistical fluctuations by random numbers/positions of dopants in Si nanovolumes^12^,^13^,^14^. These problems render conventional Si doping unsuitable for future nanoelectronics.

As an alternative to classical impurity doping, modulation doping of III-V semiconductors was introduced in the late 1970s^15^. Homostructure modulation doping was proposed for Si and Ge nanowires using conventional dopants in a Si shell around nanowires, allowing for separation of majority charge carriers from their parent donor impurities^16^,^17^. However, this approach does not solve issues of dopant inter-diffusion, deficient dopant ionization or statistical distributions of dopant number and position. An impressive work-around, referring back to junctionless FETs (Lilienfeld, 1925), was demonstrated by Colinge *et al*.^18^. This approach solves the problem of highly abrupt p-n junctions, though ultimately scaled devices again suffer from nanoscale-Si doping obstacles. Hypervalent doping of free-standing Si quantum dots was demonstrated using chemical surface engineering^19^. However, so far this approach is not at all CMOS compatible. Molecular monolayer doping^20^,^21^ was developed to achieve ultra-shallow p-n junctions via RTA-diffusion. Since this concept is based on classical impurity dopants, its applicability in low-dimensional systems is subject to the same constraints outlined above.

Here, we demonstrate a novel approach: modulation doping of Si by aluminium (Al)-induced acceptor states in silicon dioxide (SiO_2_). Using computer-aided materials design, we predict that Al atoms in SiO_2_ generate acceptor states 0.8 eV below the Si valence band edge. These states capture electrons from Si over a distance of several nanometres, providing holes to Si as majority charge carriers. We confirm this concept experimentally via capacitance-voltage (CV) and deep level transient spectroscopy (DLTS) on SiO_2_:Al/Si-based MOS capacitors. This doping technique is robust against out-diffusion, quantum confinement, dielectric confinement, and self-purification since it relocates doping from confined Si nanovolumes to adjacent bulk-like SiO_2_.

## Results

### Density functional theory simulations

**Figure 1:**
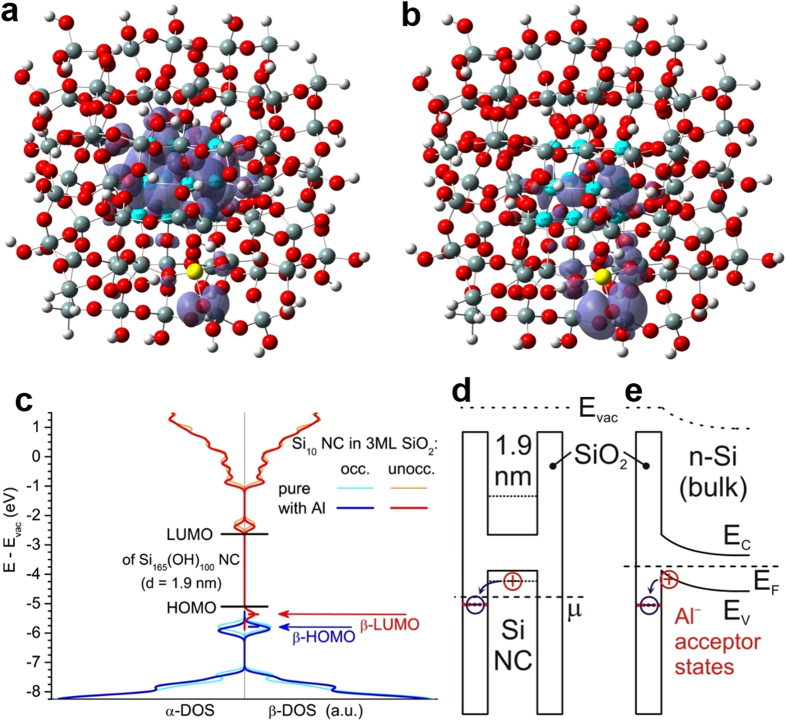
DFT results on SiO_2_ modulation doping with Al acceptors. (**a,b**) Si_10_ nanocrystal (cyan) in three ML SiO_2_ (O is red, Si grey, H white) with Al atom (yellow) replacing Si in outermost SiO_2_ shell, showing β-HOMO (**a**) and β-LUMO (**b**) as iso-density plots of 4 × 10^−4^ *e*/cubic Bohr radius. (**c**) Electronic DOS (blue, red) of that approximant (energy scale refers to vacuum level E_vac_). DOS for pure SiO_2_ embedding (cyan, orange) is shown for comparison along with the HOMO and LUMO of a 1.9 nm Si nanocrystal fully terminated with OH groups^22^. The two possible MO spin orientations are noted by α and β due to unpaired electron configuration caused by the acceptor (doublet). (**d**) Band structures showing the principle of direct modulation doping for a Si nanocrystal (NC) in SiO_2_ and (**e**) for Si bulk terminated with SiO_2_:Al layer. Occupation of electronic states are described by chemical potential μ and Fermi level E_F_, respectively.

Acceptor modulation doping of SiO_2_ was modelled via real-space density functional theory (DFT). We embedded a Si_10_ nanocrystal^22^ in three monolayers (MLs) of SiO_2_, presenting the ultimate theoretical test of the doping concept because Si nanocrystal, SiO_2_ and modulation acceptor (Al) form one approximant. Figure 1a and b show the highest occupied (HO) molecular orbital (MO) and the lowest unoccupied (LU) MO associated with Al. High probability densities occur within the Si_10_ nanocrystal and at the Al acceptor, although both are separated by 3 ML of perfect SiO_2_. This finding shows that the Si_10_ nanocrystal can still be positively ionized despite significant quantum confinement-induced bandgap widening. Figure 1c shows the electronic density of states (DOS) of the Si_10_ nanocrystals in pure SiO_2_ vs. SiO_2_:Al, along with the HOMO and LUMO of a 1.9 nm Si nanocrystal, fully terminated with hydroxyl (OH) groups, featuring SiO_2_ embedding^23^. The Al acceptor state (β-LUMO) exists 0.26 eV below the HOMO of the 1.9 nm Si nanocrystal, clearly showing that Si nanocrystals of d ≥ 1.9 nm can obtain positive majority charge carriers (holes) from Al acceptors in SiO_2_. Transferring the situation to bulk Si, the Al acceptor state is located ~0.8 eV below the valence band edge. The principle of direct acceptor modulation doping is shown in the electronic band structures in Fig. 1d for Si nanocrystals and in Fig. 1e for bulk Si.

### Electrical characterisation of modulation doped silicon

**Figure 2:**
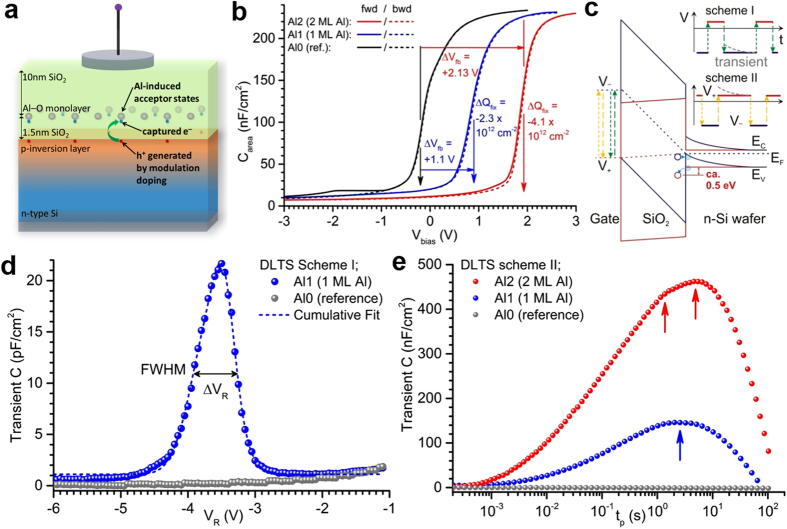
Electronic characterization of SiO_2_ modulation doping with Al acceptors using Al/SiO_2_/Al-O/SiO_2_/Si MOS structures. (**a**) Sample structure showing Al modulation acceptors charged from Si substrate by electron tunnelling. (**b**) CV curves of reference sample Al0 (no Al-O), Al1 (1 ML Al-O) and Al2 (2 ML Al-O) measured at T = 300 K; ΔV_fb_ and ΔQ_fix_ due to negatively ionized Al shown by coloured arrows. (**c**) Band structure scheme of charge transient measurements for electron release [scheme I] and for electron capture [scheme II] by Al in DLTS. (**d**) Electron release of Al in SiO_2_ with pulse time t_p_ = 205 μs, transient time T_W_ = 31 ms and pulse voltage V_p_ = +0.5 V as function of reference voltage V_R_ measured at T = 102 K to freeze out inelastic scattering and trap-assisted processes for maximum energy resolution by direct electron tunnelling into Si. (**e**) Electron capture with transient time T_W_ = 3.63 s, pulse voltage V_p_ = −4 V and reference voltage V_R_ = 0 V as function of pulse time t_p_ measured at T = 502 K to activate all transport paths (hopping, direct and trap assisted tunnelling) for maximum occupation probability of Al in SiO_2_. Arrows show sub-peaks in accord with Al-O MLs. Capacitance per area scale changes from pF/cm^2^ in (**d**) to nF/cm^2^ in (**e**).

Figure 2a shows a schematic of the Si/SiO_2_:Al MOS capacitor, fabricated via plasma enhanced chemical vapour deposition (PECVD) of SiO_2_ on n-type Si wafers combined with thermal atomic layer deposition (ALD) of 1 or 2 Al-O MLs. After deposition, the structures were annealed for 30 sec at 1000 °C in Ar ambient; subsequently, thermally evaporated aluminium contacts were structured. As evident from Fig. 1e, successful modulation doping of Si will manifest itself as a negative fixed charge Q_fix_ in the SiO_2_:Al film. Using 1 MHz high-frequency capacitance-voltage (CV), we measured a change in the fixed charge of ΔQ_fix_ = −2.3 × 10^12^ cm^−2^ for 1 ALD Al-O ML in SiO_2_ (Al1) compared with pure SiO_2_ reference samples (Al0) (Fig. 2b). The flatband voltage V_FB_ was + 0.9 V and + 1.9 V for samples Al1 and Al2, respectively, whereas for the reference sample Al0, the flatband condition was achieved for −0.2 V. By using n-type Si with a donor density of 3.5 × 10^15^ cm^−3^ and Al as the gate metal, we keep the work function difference between the gate and n-Si negligible (−0.004 eV). High-frequency CV does not allow for a contribution of Si/SiO_2_ interface states to the CV signal^24^, which can therefore be ruled out as having an effect on the CV curve. High energy resolution deep level transient spectroscopy (HERA-DLTS) was carried out to further characterize the Al-induced acceptor state. Figure 2d plots the transient capacitance over the reverse bias V_R_ at 102 K. Clearly, sample Al1 shows a dominant signal at V_R_ = −3.5 V, whereas no transient capacitance is detected for the reference sample. The peak originates from electrons escaping from the Al-state into the Si wafer via direct tunnelling (scattering, trap-assisted tunnelling and hopping are suppressed at low temperatures). The transient time constant T_W_ = 31 ms was kept comparatively short at the cost of signal intensity to further eliminate any trap-assisted tunnelling that is less dynamic. Using the Poisson equation, the energetic position of the Al-acceptor state in the SiO_2_ bandgap was calculated to be 0.5 eV below the Si valence band edge. This energy value underlines the accuracy of DFT calculations, yielding 0.8 eV. The full with half maximum (FWHM) of V_R_ was ΔV_R_ = 0.66 V, cf. Fig. 2c. The FWHM translates into a vertical-spatial distribution of Al atoms of ca. 0.27 nm, accounting for RTO interface roughness and binding of Al to the RTO, as well as binding to the CVD capping oxide.

This finding confirms our h-DFT results, where Al in SiO_2_ provides an acceptor state even for small Si NCs and corroborates that Al undergoes virtually no diffusion during the activation anneal, in accordance with its diminutive diffusion coefficient^25^. In Fig. 2e, we demonstrate the reverse pulse scheme to sample electron capture into Al acceptors and plot transient capacitance over the pulse time t_P_ at 502 K. The modulation doped sample Al1 showed one broad peak at t_P_ = 2.0 s, with a maximum transient signal of 146 nF/cm^2^ or 9.11 × 10^11^ cm^−2^ transferred charge, accounting for ca. 40% of all negative Al acceptors. No electron capture signal was detected for the reference sample. Time constants T_W_ and t_p_ were chosen to be large compared to low-temperature electron escape in order to account for the maximum density of charged Al acceptors. The substantially higher two-peak signal of sample Al2 shows that we control the density of active modulation acceptors in SiO_2_, which was confirmed by HF-CV results.

### Field effect passivation and improved hole tunnelling by Al-induced acceptor states

**Figure 3:**
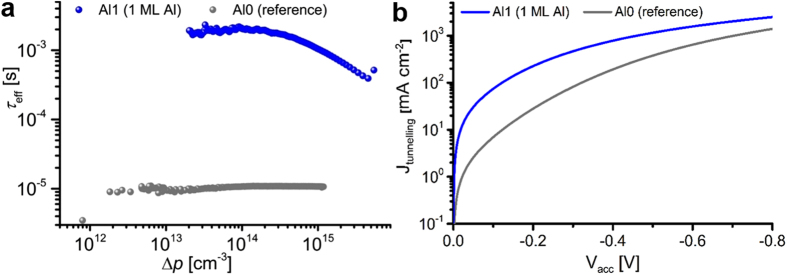
Effect of SiO_2_:Al modulation doping on effective minority carrier (hole) lifetime and hole tunnelling current density. (**a**) Double-side polished 1.6 Ωcm phosphorus-doped 525 μm Cz-Si wafers with RTO/Al-O/SiO_2_ stacks on both sides (sample Al1) show more than 2 orders of magnitude higher lifetimes compared to the RTO/SiO_2_ reference sample (Al0). The effective minority carrier lifetime of Al1 at an excess minority carrier concentration corresponding to 1 sun illumination (Δp = 10^15^ cm^−3^) is τ_hole_ = 1 ms. (**b**) Hole tunnelling current density under accumulation bias on boron-doped Cz-Si wafers with RTO/Al-O/SiO_2_ stacks (3 nm total thickness). The Al-O monolayer in sample Al1 enables 1 order of magnitude higher hole current densities at small bias as compared to sample Al0 (RTO/SiO_2_ reference sample).

First results on carrier lifetime measurements carried out by quasi-steady-state photoconductance (QSSPC)^26^ are shown in Fig. 3a using mechanical grade double side polished 525 μm thick Cz-Si wafers with an ultrathin rapid-thermal oxide. Samples were not passivated in hydrogen (H_2_) or forming gas (FG). The spectra show a minority (hole) carrier lifetime τ_hole_ = 1 ms at an excess majority carrier density corresponding to 1 sun illumination (Δp = 10^15^ cm^−3^), presenting an increase over the reference sample with undoped annealed CVD-SiO_2_ by a factor of 100. The corresponding emitter saturation current density^27^ is j_0_ = 1.05 × 10^−13^ A/cm^2^. This low value obtained on preliminary samples shows the potential of SiO_2_ modulation doping for high efficiency silicon solar cells. With a high negative Q_fix_ we can approximate the surface recombination velocity by^28^ S_eff_ = d_wafer_/2τ_hole_, yielding 26 cm/s. In Fig. 3b current-voltage measurements on 3 nm thick RTO/SiO_2_ stacks on p-type Si wafers are shown. Current densities of ca. 40 mA/cm^2^ which correspond to maximum power point (MPP) operation of high efficiency solar cells incur a potential drop of ca. 0.23 V for the reference sample while the SiO_2_ doped with 1 ML Al reduces this potential drop by 74% to 0.06 V. While a high negative Q_fix_ accelerates holes to the tunnelling barrier, the Al-induced acceptor states do not have to be charged for increasing the transition probability through the tunnelling barrier. Such states contribute to improved hole tunnelling already in their neutral form due to diminishing the tunnelling barrier from the valence band offset from Si to SiO_2_ of ca. 4.5 eV^29^ to merely 0.5 eV by introducing a lateral defect band. The improved lifetimes and tunnelling currents obtained for preliminary, non-optimized Cz-wafers indicate the promising potential of SiO_2_:Al for passivating hole selective tunnelling contacts in high efficiency silicon solar cells.

## Discussion

**Figure 4:**
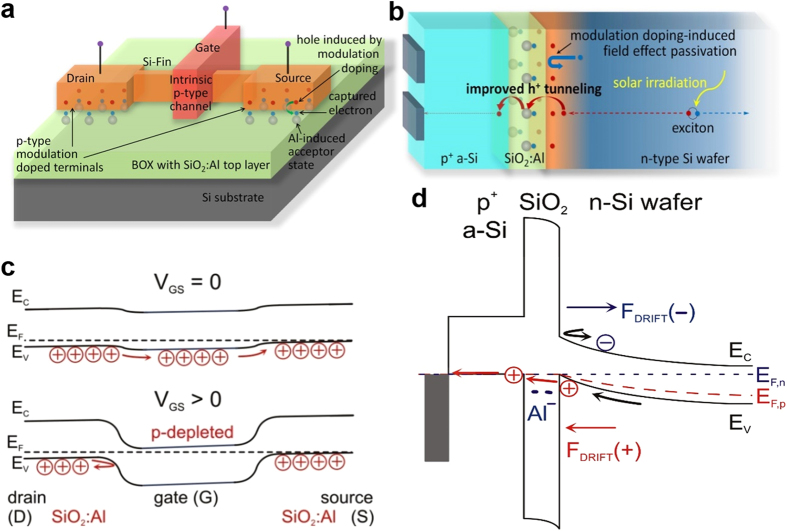
Application examples for Si modulation doping. (**a,c**) Undoped Si fin-FETs can be provided with holes as majority carriers from Al acceptors in the buried oxide layer forming the base of the fin (**a**), eliminating any size limit due to conventional doping. The band diagram of such fin-FET (**c**) shows its working principle as hole depletion (p self-conducting) transistor. (**b,d**) HIT Si solar cells can be equipped with massively enhanced hole contacts where SiO_2_ is the optimum choice in terms of chemical bond interface passivation (**b**). Moreover, SiO_2_ provides a much increased minority (electron) barrier while accelerating holes through a low tunnelling barrier thanks to negatively charged acceptors located about 0.5 eV below the valence band of c-Si. The band diagram of such a hole contact shows its working principle to provide much increased conversion efficiencies of HIT solar cells (**d**).

Heterostructure modulation doping of Si using intentionally designed impurities in SiO_2_ presents a true paradigm shift by “outsourcing” dopants to the surrounding matrix. This approach can acceptor-dope Si MOS-FETs by incorporating Al atoms in the insulating SiO_2_ trench or into the base and coating for fin-FETs, which circumvents all mentioned nanoscale doping problems. As shown exemplarily in Fig. 4a,c, the source and drain areas of an intrinsic Si fin-FET become p-type conductive by incorporating Al-atoms in the buried oxide (BOX). The absence of dopants within the active Si volume eliminates dopant impurity scattering, resulting in lower heat generation, higher carrier mobilities and consequently lower operating voltages. These features are very beneficial for advancing ultra-large scale integration (ULSI) and ultra-low power applications. Moreover, modulation doping avoids the so-called dopant fingerprint of nanoscale MOS-devices, i.e. statistical performance fluctuations due to variations in the exact number and distribution of dopants^4^,^12^,^14^. Coulomb repulsion between the charged Al-induced acceptor states self-regulates the amount of holes generated and prevents that all such states capture an electron. In fact, approximately only one in a hundred acceptor states (1 Al-O monolayer) can be charged and hole-doping is easily controlled via the areal coverage of the Si nanovolume with SiO_2_:Al.

Apart from microelectronics, Si-modulation doping can enhance passivating tunnelling contacts in heterojunction with intrinsic thin layer (HIT) solar cells^30^, cf. Fig. 4b,d. Very recently, the conventional HIT-cell concept was complemented by the DASH cell concept (dopant-free asymmetric heterocontacts)^31^. Bullock *et al*. propose alkali metal fluorides such as LiF_x_ on intrinsic a-Si:H as electron selective heterocontacts^31^ and MoO_x_/a-Si:H(i) as hole selective heterocontacts^32^ and achieve impressive conversion efficiencies. In a different approach, electron-selective contacts were realized by ALD-TiO_2_ on ultrathin tunnel-SiO_2_^33^.

We propose that the Si modulation doping approach represents a competitive technology to improve efficiencies even further: Modulation acceptors in ultrathin SiO_2_ significantly improve hole tunnelling transport via a strongly decreased tunnelling barrier (0.5 eV vs. 4.5 eV in undoped SiO_2_), cf. Fig. 3b. Additionally, the acceptor states provide a negative drift field for repelling electrons as the minority carrier type, complemented by the unparalleled chemical Si surface passivation quality of SiO_2_. A massive increase in minority carrier lifetime and ensuing ultra-low emitter saturation current density as compared to unpassivated samples is evident from Fig. 3. Currently used amorphous Si surface layers are far from featuring such properties. Interestingly, a recent theoretical screening study investigated several elements concerning their defect state energies in SiO_2_ with respect to Si, in order to improve field-effect passivation and transport for majorities^34^. However, the energetic position derived for Al in that study differs vastly from our theoretical DFT and experimental DLTS values.

Finally, we note that one or two Al-O monolayers in SiO_2_ do not constitute an Al_2_O_3_ phase. Field effect passivation by negative Q_fix_ were also demonstrated with Al_2_O_3_ films prepared by pyrolysis^35^, sputtering^36^, PECVD^37^, and ALD^38^,^39^,^40^,^41^. These stacks often require annealings in the range of 400 to 800 °C to build up the negative Q_fix_, depending on the deposition technique. Remarkably, it was shown that 1 to 2 nm silicon oxide inevitably grow during deposition and annealing and that the negative charges are majorly located at the SiO_2_/Al_2_O_3_ interface^38^,^39^. We hypothesize that Al_2_O_3_ undergoes an interface reaction with underlying Si most likely during the anneal to form an ultrathin layer of SiO_2_, thereby creating O-deficient Al_2_O_3_ (Al_2_O_x_, x < 3) which contains O vacancies. This situation is in striking analogy to F-deficient aluminium fluoride (AlF_x_, x < 3) as mentioned below^42^,^43^. In one work^39^ the origin of the negative Q_fix_ is ascribed to *excess* oxygen (O) within AlO_x_ (i.e. x > 3). Since O is very reactive and either a radical (monatomic form) or a gas (O_2_) and the respective samples require an activation anneal for 15 min at 425 °C^39^, it is inconceivable how excess O could exist in such AlO_x_ layers. As a result, we have reasonable doubt to consider excess O as the origin of the negative Q_fix_. On the other hand, average values of Q_fix_ = −4 × 10^12^ cm^−2^ and midgap Si/SiO_2_ interface trap densities of 3 × 10^10^ cm^−2^ measured by mercury-probe CV (Hg-CV) wafer maps have been routinely obtained for F-deficient AlF_3_ (AlF_x_) on Si wafers with ultrathin SiO_2_^42^. Light soaking increases the average value of this negative Q_fix_ to −4.85 × 10^12^ cm^−2^ 43. DFT calculations show that F vacancies are acceptor type defects which can localize electrons originating from Si and tunnelling though SiO_2_^42^,^43^,^44^,^45^, yielding a negative Q_fix_. We note here that O was not present in AlF_x_ nor did the ultrathin SiO_2_ layer on the Si wafer contain excess O^43^. These findings clearly support the picture that O vacancies provide the acceptor type defect states in AlO_x_ manifesting the negative Q_fix_. In contrast, modulation acceptor states in SiO_2_:Al owe their existence to *one O dangling bond next to a single Al atom which substitutes Si in SiO*_*2*_, see Fig. 1a–c. Although Al_2_O_3_ thin films provide field effect passivation in analogy to Al-doped SiO_2_ on Si, the insulating nature of Al_2_O_3_ in compound with the 2 nm SiO_2_ layer formed during the anneal prevents improved hole tunnelling as demonstrated here for 1 Al-O monolayer in SiO_2_, cf. Fig. 3b. Doping SiO_2_ with Al also eliminates oxidizing interface reactions whereby sample processing becomes more controllable which can be a technological advantage. The performance of Al_2_O_3_ was also investigated in the limit of 1 and 4 plasma ALD-cycles and corresponding thicknesses of 1 and 5 Å^40^,^41^. The thickness of 1 Å corresponds to one thermal ALD cycle as used by us to dope SiO_2_ with 1 ML Al-O. It turns out that these ultrathin films deposited directly on Si create significantly less negative Q_fix_, though a superposition with the high positive fixed charge density in the SiN_x_ capping cannot be ruled out.

In summary, we developed in theory and experiment a heterostructure Si-modulation doping method based on aluminium-induced acceptor states in SiO_2_. Providing holes as majority charge carriers with such a fundamental principle represents a paradigm shift in silicon science and technology. It allows for a different strategy to define and control majority charge carriers in nanoelectronic devices and allows for passivating hole-selective contacts in high-efficiency solar cells. More generally, our modulation doping concept is transferable to other group-IV semiconductors, such as diamond or germanium, if energetically suitable combinations of impurity elements and dielectric matrices are found.

## Methods

### Density functional theory simulations and Poisson solver

Approximants were structurally optimized for the maximum integral over all bond energies defining the most stable configuration, using the Hartree-Fock (HF) method with 3-21G molecular orbital basis set (MO-BS)^22^,^46^ for structural optimizations and the B3LYP hybrid DF^47^,^48^ with 6–31G(d) MO-BS^22^,^49^ for electronic structure calculations with the Gaussian09 software suite^50^. RMS and peak force convergence limits were 8 meV/Å and 12 meV/Å, respectively. Ultrafine integration grids and tight convergence criteria were applied to the self-consistent field routine. During all calculations, no symmetry constraints were applied to the MOs. Further accuracy evaluations can be found elsewhere^22^,^23^. A one-dimensional (1-D) Poisson solver for MIS structures was coded in MatLab following Nicollian and Bruce^24^.

### Fabrication and characterization of SiO_2_:Al samples

PECVD SiO_2_ tunnel oxides (1.5 nm) were deposited^51^ onto wet-chemically cleaned 100 mm Czochralski P-doped Si wafers (1.6 Ωcm) in a modified Oxford Instruments PlasmaLab–FlexAL cluster. Subsequently, the wafers were transferred under vacuum into the thermal ALD chamber for deposition of Al-O monolayers via TMA (trimethylaluminium) and H_2_O at 200 °C (sample Al1: 1 ALD cycle, Al2: 2 ALD cycles, Al0: H_2_O pulse only). Finally, the capping SiO_2_ layer (10 nm) was deposited in the PECVD chamber. The lifetime wafers were rapid-thermally oxidized (RTO, 2.5 nm) and then transferred into the ALD-PECVD cluster for Al-O monolayer and capping oxide deposition. All wafers were RTP-annealed (1000 °C, 30 s, Ar atmosphere). Electrical top- and substrate contacts (Al) were thermally evaporated and lithographically structured to fabricate MOS capacitors. The hole tunnelling samples were fabricated similarly on B-doped Cz-Si wafers (2.4 Ωcm) with 1.5 nm RTO and capping oxide to enable direct tunnelling and thermally evaporated Pd contacts. All silicon oxide thicknesses were measured by ellipsometry. DLTS and HF-CV were measured with a PhysTech FT1030 High energy resolution analysis (HERA) setup using a JANIS VPF 800 Cryostat. I-V was measured with an Agilent B1500A. Minority (hole) carrier lifetime was measured as average value over the entire wafer with QSSPC using a lifetime tester from Sinton Consulting.

## Additional Information

**How to cite this article**: König, D. *et al*. Modulation Doping of Silicon using Aluminium-induced Acceptor States in Silicon Dioxide. *Sci. Rep.* **7**, 46703; doi: 10.1038/srep46703 (2017).

**Publisher's note:** Springer Nature remains neutral with regard to jurisdictional claims in published maps and institutional affiliations.
